# Undescended ovary and fallopian tube presenting as appendiceal mucocele

**Published:** 2018-03

**Authors:** Pieter Sinonquel, Julie Bontinck, Marianne Stevens

**Affiliations:** UZ Leuven, Department of Internal medicine, Herestraat 49, 3000 Leuven, Belgium; Sint-Jozef Kliniek, Department of general and oncological surgery, Kasteelstraat 23, 2880 Bornem, Belgium; Sint-Jozef Kliniek, Department of gynecology and obstetrics, Kasteelstraat 23, 2880 Bornem, Belgium.

**Keywords:** congenital uterine malformations, ectopic ovary, infertility, uterus unicornis, undescended ovary

## Abstract

Undescended ovary is a rare congenital gynecologic condition, frequently associated with urogenital malformations including unicornuate uterus and ectopic kidneys or renal agenesis. Although ectopic ovaries are mostly found during an infertility work up, its role in infertility is still unknown. We report a 38-year-old patient presenting with pain in the right lower quadrant. Explorative laparoscopy reveals a unicornuate uterus and a malpositioning of the right ovary and distal ending of the right fallopian tube. Through this report, we try to provide hints for guiding the diagnostic management of similar patients in terms of fertility, renal function and tumour formation.

## Introduction

To understand the phenomenon of undescended ovaries, a good comprehension of the embryological development is primordial. Gonads develop during the fifth week of pregnancy as a cluster of proliferating cells at the medial side of the urogenital ridge (Trinidad et al., 2004). At this stage ovaries and testes have an analogous development. At the same time the mesonephric (wolffian) duct and the paramesonephric (mullerian) duct develop (Jacquinet et al., 2016). At 6 weeks the gonadal cells differentiate into leydig and sertoli cells in the male foetus, whereas in the female foetus the formation of follicles starts at 10-11 weeks of pregnancy. The first primordial follicles develop at the age of 16 weeks (Schoenwolf et al., 2009). In males the leydig cells produce testosterone, inducing the differentiation of the Wolffian duct into the male internal sex organs (epididymis, seminal vesicles and ductus deferens). The male sertoli cells produce antimullerian factor (AMF), which initiate the degradation of the mullerian duct (Pask, 2016). Since females produce estrogen instead of testosterone and do not produce AMF, the wolffian duct degrades and the mullerian duct forms the later female internal sex organs (uterus and fallopian tubes) (Jacquinet et al., 2016). During the third month of foetal life, the gonads descend from their initial location near the kidneys towards the pelvis guided by chemotactic factors. Testes descend further through the inguinal canal into the scrotum, whereas ovaries rest intra-abdominal beneath the pelvic brim. This descent is guided by the gubernaculum, a string of mesenchymal tissue attached to the inferior pole of the gonad. At final stage, the cranial gubernaculum forms the round ligament of the ovary and the caudal part becomes the round ligament of the uterus, which runs through the inguinal canal and ends in the labia majora. Together they make the broad ligament of the uterus. The ovarian suspensory ligaments are attached at the superior pole of the foetal ovary and become the infundibulopelvic ligament (Schoenwolf et al., 2009; Parmley 1993). If one phase of this multistep process is altered, the gonadal descent might be disturbed and the gonads will probably be dislocated. The underlying process of ovary maldescent has still to be elucidated. Some claim it could be resulting of a lack of caudal descent or due to a specific gonadal growth restriction (Parmley, 1993; Van Hooris et al., 2000). Undescended testes are far more frequent than undescended ovaries, since they pass through the narrow inguinal canal. The prevalence of maldescended ovaries is estimated 0,3 – 2% (Verkauf et al., 1996). Ovarian maldescent is rarely found in association with a normal uterus. The anomaly of ovarian and fallopian maldescent are more frequently seen in combination with uterine anomalies as up to 42% with a unicornuate uterus. The rare Mayer-Rokitansky-Küster-Hauser Syndrome (MRKH) is diagnosed in 20% of the patients with uterine anomalies in combination with ectopic ovaries (Parmley, 1993). Although the link between undescended ovaries and unicornuate uterus is well known, undescended ovaries are infrequently reported, hence suggesting a possible under diagnosis of this anomaly (Verkauf et al., 1996; Ombelet et al., 2003b; 2003c; 2011). Ectopia of the ovary may also be accompanied by concomitant maldevelopments of the genital tract which frequently are accompanied by anomalous changes in the renal system. Renal agenesis is a relatively common congenital anomaly, although its etiology is unknown. This agenesis may be unilateral or bilateral and is generally thought to result from: 1. Absence of the metanephric blastema; 2. Ureteral bud maldevelopment; or 3. Lack of induction of the metanephric blastema by the ureteral bud. Moreover, renal agenesis occurs when the ureteric bud fails to branch in combination with failure of nefron development. Unilateral renal agenesis is usually an incidental finding. Renal agenesis is also associated with ipsilateral urogenital anomalies (Kollia et al., 2014). Furthermore, ectopic ovaries are known to cause menstrual irregularities, infertility or abdominal pain.

## Case description

A 38-year-old nulliparous woman presented at our emergency unit with right lower abdominal pain. Biochemically there were no changes worth mentioning. She had a negative pregnancy test, normal C-reactive protein (CRP) and no other signs of infection.

**Figure 1:**
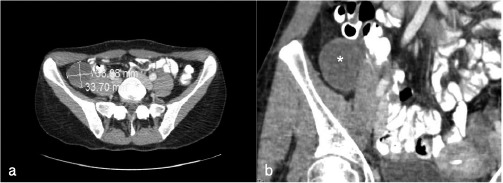
— Figure 1a-1b: A 38-year-old woman with right fossa syndrome. a. Coronal computed tomography (CT) image of the abdomen showing a cystic formation in the right flank (33,70 x 38,08 mm). b. Frontal CT image sowing a cyst of 40 Hounsfield Units (asterisk).

Abdominal ultrasound showed a non-echogenic cyst in the right flank. Computed tomography (CT) scan of the abdomen with intravenous and oral contrast confirmed a right lower abdominal cystic formation with a diameter of approximately 38 by 33 millimetres (Fig. 1a) and a density of 24 Hounsfield units (HU) (Fig. 1b). Imaging also revealed a narrow approximation of the cyst with the colon ascendens and the psoas muscle as well as a very discrete infiltration of the perilesional fat tissue. The differential diagnosis of enteric duplication cyst, mesenterial cyst, peritoneal inclusion cyst and appendiceal mucocele was established. Gynecological examination showed a normal cervix in speculo. Bimanual vaginal examination reported a mobile uterus with normal adnexa. Vaginal ultrasound showed an intra uterine device in the uterine cavity and a normal left ovary. The right adnex could not be visualized. To further investigate the origin of the abdominal pain, the patient was hospitalized and planned for an explorative laparoscopy a few days after the first symptoms appeared.

**Figure 2:**
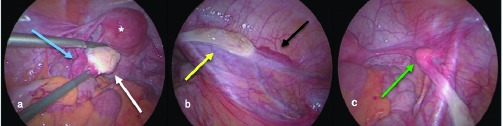
— Figure 2a-c: A laparoscopic view of the abdominal and pelvic organs. a. Unicornuate uterus (white asterisk), normal left ovary (white arrow) with the fallopian tube (blue arrow). b. Left ectpoic ovary (yellow arrow) and fimbral ending of the right fallopian tube (black arrow). c. Firm spherical formation (green arrow), believed to be a rudimentary part of the unicornuate uterus.

During laparoscopy, the appendix was found to be normal, without mucocele. The cecum was located deep in the right fossa. Exploration of the internal genitals showed a unicornuate uterus and a normal left fallopian tube and ovary (Fig. 2a). The right fallopian tube and ovary could not be visualized in the right pelvis but were found to be infrahepatically. Both the ovary and fallopian tube were positioned upon the psoas muscle, which was also crossed over by the right ureter. (Fig. 2b) Over the course of the left round ligament we observed a firm spherical formation, most likely a rudimentary part of the unicornuate uterus (Fig. 2c). In summary, we concluded that this was an anatomical anomaly of the right mullerian system, known as an infrahepatic undescended ovary with adjacent fimbrial ending of the fallopian tube. The cystic formation seen on abdominal CT could not be visualized during laparoscopy. We believe it may have been a temporary follicular cyst of the smaldescended ovary.

## Discussion

Ovarian maldescent or ectopic ovary is a rare embryological malformation, mostly found by coincidence during an infertility work up and known under different names. Lachman and Berman suggested a classification of undescended ovaries in 1991: 1. Post-surgical implant, 2. Post-inflammatory implant, and 3. True (ectopic) ovarian tissue (Lachman et al., 1991). Maldescended ovaries have a very low incidence, between 0,3% and 0,5%. Its prevalence increases in women with a unicornuate uterus, rarely seen with bicornuate and didelphys uterus (Trinidad et al., 2004; Dietrich et al., 2007). It can be unilateral or bilateral (Trinidad et al., 2004). The low incidence might be due to under diagnosis, since ectopic ovaries can be easily missed during standard gynecological work-up. Most diagnoses are established during infertility exploration including hysterosalpingography or magnetic resonance imaging (MRI) after clomiphene citrate stimulation (Ombelet et al., 2003b). As in our case, explorative laparoscopy is another possible diagnostic tool and remains the gold standard to reveal the cause of ovarian pathology. The benefits of MRI are 1. It is a non-invasive, highly sensitive and specific radiologic modality 2. Concurrent diagnosis of renal anomalies possible and 3. Follow-up in patients with infertility or recurrent pelvic pain is easily possible (Litos et al., 2003; Kollia et al., 2013).

Unicornuate uterus and associated urinary tract anomalies including renal agenesis, ectopic kidneys, horseshoe kidney and double renal pelvis are well known. The investigation of the kidney function, their presence and morphology via intavenous pyelography is primordial in patients with ectopic ovaries. In our case, two morphologically normal kidneys were found on CT scan with a biochemically normal glomerular filtration rate. No further renal investigations were done. Renal agenesis and a unicornuate uterus can also be part of the MRKH-syndrome (Parmley, 1993). This rare congenital gynecologic disorder is characterized by hypoplasia of the uterus and upper part of the vagina, classically presenting with primary amenorrhea (Miao et al., 2018). In 20% of the reported cases ectopic ovaries were found. In terms of fertility, undescended ovaries and a non-communicating fallopian tube have little risk of ectopic pregnancy. Although we do not know if ectopic ovaries are more frequent in patients with an ectopic pregnancy, since only a few cases have been reported until now (Trinidad et al., 2004; Seoud et al., 1987). Ombelet et al. described a normal intra-uterine pregnancy in a patient with an undescended ovary due to transperitoneal oocyte and sperm migration (Ombelet et al., 2003a). A significant association between undescended ovary, associated fallopian tube and infertility has not been shown (Verkauf et al., 1996). Due to the limited amount of reports and possible publication-bias, further investigations are needed to draw definite conclusions about the relationship between infertility and ectopic ovaries. Since cryptorchidism is a risk factor for gonadal tumour formation, undescended female gonads may as well have a higher risk of tumour development. Subramony et al. reported 12 cases of primary retroperitoneal cystadenomas. (Trinidad et al., 2004; Subramony et al., 2001). Whether these neoplasms are actually tumours of undiagnosed maldescended gonads needs to be further evaluated.

In conclusion, we present a rare case of a unilateral ectopic ovary and unicornuate uterus in a 38-year-old patient with pain in the right lower quadrant mimicking an appendiceal mucocele. We believe this mucocele to be a temporary follicular cyst of the maldescended ovary. Therefore, we tried providing clinically relevant hints for guidance in the diagnostic management of similar patients.
